# What can surgery learn from other high-performance disciplines?^☆^

**DOI:** 10.1016/j.amsu.2020.04.007

**Published:** 2020-04-24

**Authors:** Jessica O'Logbon

**Affiliations:** GKT School of Medical Education, King's College London, Hodgkin Building, Newcomen St., London SE1 1UL, UK.

**Keywords:** Performance, Training, Surgery, Error, Patient safety

## Abstract

High-performance disciplines have always been concerned with safety and exceptional performance. They have established a culture of vigilance and accepted that human error is both inevitable and ubiquitous. These disciplines, therefore, have all implemented a ‘systems approach’ to error by focusing on predicting, preventing, rescuing and reporting errors that occur so that they can constantly adapt and improve. Given the complexity of surgery, and the error-prone environment within which it takes place, extracting positive behaviours from other high-performance disciplines will serve to improve performance and enhance patient safety. Surgery is being practiced in an ever-changing environment. Currently, there is less available operative experience for surgical trainees; multi-morbidity in patients is growing and rapidly evolving technology means that more high-tech equipment is being used in procedures. This article evaluates the effectiveness of current surgical protocol in reducing errors and possible modifications that can be made to fit the new environment that surgery is now being practiced in. It will then describe how three different high-performance disciplines: aviation, professional sport and Formula 1, have developed in their approaches to safety and excellence, which will serve as the basis for a discussion about what more can be learnt from these disciplines so that the surgical profession can continue to excel in the face of change.

## Highlights

• High-performance disciplines must strive for error-free performance.
• Surgical protocols need to be adapted to fit the dynamic surgical environment.
• Surgery can adopt new strategies from aviation, Formula 1 and professional sport.
• Coaching, video analysis of performance and benchmarking can transform surgery.

## 1 Introduction

Aviation, Formula 1 and professional sport, though seemingly different, are examples of high-performance disciplines. Each has the potential for catastrophic failure yet achieves nearly error-free performance. What makes these disciplines unique is their commitment to safety at the highest level and their specialist approaches to its pursuit [1]. The surgical profession has already adopted some strategies from aviation and Formula 1 (F1) including checklists, team training initiatives and simulations, with demonstrated benefits in team performance and reductions in medical errors [[2], [3], [4], [5]]. However, surgery is being practiced in an ever-changing environment and safety improvement strategies must continuously adapt. At present, there is less available operative experience during surgical training [6]; high-tech equipment is being used in procedures more than ever before [7]; and an increasing number of patients are multimorbid [8]. This article evaluates the effectiveness of current surgical protocols and suggests ways that they can be modified to fit the new, dynamic surgical environment. Methods that can be extrapolated from aviation, F1 and professional sport in order to continue to reduce errors and drive excellence are discussed and what this will mean for the future of surgery.

## 2 What surgery has already learned

The surgical profession has begun to follow the lead of industries like aviation and F1 in their approaches to safety and performance. The seamless teamwork and coordination demonstrated by F1 pit-stop crews has been emulated in surgical practice to reduce handover errors [5]. In addition, surgery has applied the theories of aviation crew resource management (CRM) to promote teamwork and communication among care teams and checklists to reduce the risk of adverse events [9,10].

Adverse events account for significant morbidity and mortality and it was found that almost half of these events were preventable in surgery [[11], [12], [13], [14]]. Furthermore, a systematic review including hospitals in the USA, UK, Canada, Australia and New Zealand, found that most adverse events were associated with a surgical care provider (58.4%) and the majority of these were operation-related (39.6%) [15]. On closer examination, many adverse events classified as ‘operation-related’ are actually found to be due to problems in ward management rather than intraoperative care [16]. This demonstrates that patient safety improvement efforts should not only target errors in surgical technique but critically focus on post-operative management.

The implementation of surgical safety checklists has reduced common intraoperative errors (such as wrong-site surgery and incorrect anaesthesia), increased communication and teamwork and led to a decline in mortality rates [2,17]. Despite these known benefits, it is important to acknowledge that successful checklist usage requires high compliance rates and must be appropriate for the environments in which they are implemented since the type and frequency of errors are not homogenous throughout all surgical specialties. For instance, equipment-related errors occur more often in specialties that rely heavily on technology such as orthopaedic surgery [18]. Therefore, these checklists should include items addressing the main categories of equipment problems: availability, configuration and settings and malfunctioning/failure then modified to reflect the technology of the procedure [19]. Checklists also represent a low-cost intervention for standardising postoperative care; enhancing the quality of ward rounds and allowing for early identification and management of postoperative complications.

Furthermore, following a public inquiry into the high mortality rates for paediatric cardiac surgery, Ferrari helped Great Ormand Street Hospital to develop a 4-stage handover process and adopt the methodology of the ‘lollipop man’ (the anaesthetist) to coordinate this [20]. The F1 pit‐stop crew was a model example of how a multi‐professional team works as a single unit to perform a complex task under huge time pressure with minimal error. Streamlining of the preoperative process, treatment and recovery was optimised through standard operating procedures with a multidisciplinary team where all stakeholders were actively engaged, but this is not consistent practice. On weekends, staff are often stretched and unfamiliar with the patients under their care and a structured, comprehensive electronic handover system could provide better continuity of care than verbal/written handovers [21].

Non-Technical Skills Training for Surgeons (NOTSS), modelled on aviation CRM, was introduced in 2006 and has resulted in improved attitudes to safety, team performance and technical error rates both in the operative field and outside it [4,22,23]. With these improvements in safety, such courses should be formally embedded into all medical and nursing schools' standard curricula to improve students’ handling of emergencies. A curricular design that combines regular practical simulation sessions with debriefings and human factors seminars appears to be crucial [24]. Regular assessments (of individuals and teams) are also necessary to change workplace culture and stakeholder attitudes in order to foster long-term benefits.

## 3 The Cockpit vs. The operating room

Cockpit behaviour is analysed through routine use of simulators and Line Operations Safety Audits (LOSAs) [25]. LOSAs involve audio-visual recordings of aviation staff in live flight; an approach that can be applied to the assessment of surgical team functioning in the operating room. Direct observation enables the reliable assessment of non-technical skills for each individual in the team, as well as how team functioning changes with case complexity, stress level and distractions. Such assessments with human observers have been piloted and errors in surgical technique had a strong association with situational awareness [26,27].

However, this approach has only been validated in general surgery and, because of the complex nature of observing and detecting human behaviour, it is plausible that there may be observer bias and discrepancies in scoring amongst observers from different training backgrounds. Moreover, as the Hawthorne effect may artificially raise performance standards in short-term observational studies, taking a continuous monitoring approach parallel to LOSAs may prove more effective [28]. To date, video-analysis of technical skills has demonstrated the additional educational value of video recording in a range of specialties but is not often used for non-technical skills [29]. A range of ethical, legal and financial restrictions may account for this, such as patient consent and the cost of multiple audio-visual devices to capture all of the operating room being assessed. Nevertheless, such a tool is highly valuable to benchmark good teamwork skills and guide formative feedback and debriefing in clinical practice, which will ultimately save lives.

Simulation has the potential to prepare trainees to manage a range of scenarios from basic procedures to intraoperative complications, rather than refining their technical skills in real-life situations. Virtual Reality (VR) and Augmented Reality (AR) are propelling surgery towards this [30]. The ability to reproduce the complexity of human anatomy and obtain realistic simulation haptics would allow surgeons to train in complex 3D anatomical environments with realistic tissue and organ behaviour [31]. Current evidence suggests that basic surgical skills acquired on simulators can be transferred to the clinical setting [32,33] therefore, developing an educational curriculum that incorporates regular simulation could begin to tackle the reduction in operative experience available for surgical trainees and reduce the chance of patient harm.

## 4 The surgeon and the professional sportsperson

Professional athletes are successful due to natural ability and hard work but even the most elite turn to coaches to ensure they continue to perform their best. Should surgeons?

Surgeons spend over a decade acquiring technical skills and refining these under the supervision of more experienced colleagues, though what happens after completing this formal training is far less rigorous. Since traditional teaching is based on a presumption that after a certain point the student no longer needs instruction, surgeons are responsible for maintaining and improving their own skills for most of their careers. Despite this, current strategies for professional development are suboptimal and without the opportunity for longitudinal learning from other talented colleagues, surgeons risk plateauing as their careers progress.

Coaching is one promising approach to tackle this. The GMC and the foundation programme curriculum identify coaching and mentoring as “essential to supporting and developing good practice” [34,35]. Detailed monitoring of performance allows a coach to give constructive feedback, facilitate self-reflection, guide action planning, and support implementation and self-evaluation of changes in practice, which plays an integral part in the success of athletes [36,37]. Evidence to support the effectiveness of surgical coaching is in its early stages but is growing quickly, with studies showing that it enhances learning and results in better skill acquisition than conventional training for both trainees and practicing surgeons across a range of contexts [38,39].

A national coaching programme for UK trainees does not currently exist but has great potential to become a standard component of surgical practice. The growing pool of retirees represents a reserve of accumulated experience and near-peer surgical coaching relationships represent a useful approach in specialties with rapidly evolving technologies such as orthopaedics, urology and ENT.

Furthermore, with improving remote connectivity, technology like AR has allowed surgical experience to be distributed across the world via platforms such as Proximie, paving the way for ‘tele-mentoring’. In the UK, a single junior member of staff will often be responsible for out-of-hours surgery and tele-mentoring could enable them to share a real-time view of difficult cases with an off-site senior surgeon for immediate advice, reducing the likelihood of clinical error and unnecessary patient transfers [40].

## 5 Benchmarking in Formula 1

F1 is a data-driven sport. Every variable of the car, from aerodynamics to tyre pressures, is monitored and analysed. The data collected is then used to establish a target performance level (benchmark) – informing driver tactics and prompting F1 teams to change strategy and redesign the car week-by-week to beat the competition. In healthcare, establishing benchmarks has been less specific, where comparisons often do not target the best but average results. With a lack of objective data on surgical performance and comparison with peers and best practices, surgeons cannot determine whether their efforts are satisfactory or exceptional, and more importantly, what needs improvement.

A shift has occurred over the past decade with increasing focus on morbidity instead of mortality in surgical outcomes, and there continues to be expansion and validation of Patient Reported Outcome Measures [41]. Therefore, new benchmarks must be defined for every type of operation, incorporating patient-specific factors, and continually updated. This means that future strategy will rely on national and international databases – requiring surgeons to participate in regular audits. Despite potentially large improvements in patient safety, major challenges to this approach include the time and labour-intensive collection of data, the potential for inaccuracy, and balancing competing ideals of achievability and perfectionism.

Artificial intelligence (AI) is becoming increasingly used by high-performance disciplines to predict risk, allowing crises to be averted and outcomes optimised. In surgery, AI analysis of ‘Big Data’, as seen in F1, could allow the automated transmission of pre- and post-operative mobile data (e.g. health apps and fitness trackers) directly into Electronic Health Records. This could provide a more patient-specific risk score for operative planning and yield valuable predictors for postoperative care [42,43]. Interventions could then be undertaken to reduce risk at every stage of the patient journey: targeted ‘prehabilitation’ could be employed to optimise modifiable risk factors before surgery; operative techniques and monitoring could be tailored to the patient's needs, balancing safety with efficacy; personalised post-operative monitoring and care could be utilised to enable effective resource allocation and potentially reduce the harm of post-operative complications by early detection and prevention.

## 6 Conclusion

The surgical field has already utilised concepts developed in other high-performance industries with positive effects on patient safety and team performance. Yet such systems need further adaptations to suit the ever-changing environment that surgery is being practiced in. In professional sports, coaching provides continual analysis and feedback on performance which underpins the success of athletes and should be formally introduced into surgical practice. Regular video analysis of team functioning in the operation room can monitor and refine fundamental technical and non-technical skills. The NHS must optimise benchmarks for operations, incorporating patients' factors and national operative registries. Effective use of ‘Big Data’ has the potential to optimise patient outcomes and minimise risk, whilst VR & AR can be used to improve surgical performance and safety. Employed correctly, these methods can lead to better efficacy, efficiency and safety in surgery.

## Ethical approval

Nothing to declare.

## Sources of funding

Nothing to declare.

## Author contribution

Nothing to declare.

## Reasearch registration number

1. Name of the registry: N/A.
2. Unique Identifying number or registration ID: N/A.
3. Hyperlink to the registration (must be publicly accessible): N/A.

## Guarantor

Jessica O’Logbon.

## Provenance and peer review

Not commissioned, externally peer reviewed.

## Declaration of competing interest

None.
